# RETRACTED: El-Garawani et al. *In Vitro* Induction of Apoptosis in Isolated Acute Myeloid Leukemia Cells: The Role of *Anastatica hierochuntica* Methanolic Extract. *Metabolites* 2022, *12*, 878

**DOI:** 10.3390/metabo15080544

**Published:** 2025-08-12

**Authors:** Islam M. El-Garawani, Amira S. Abd El-Gaber, Noura A. Algamdi, Aamer Saeed, Chao Zhao, Omar M. Khattab, Mohamed F. AlAjmi, Zhiming Guo, Shaden A. M. Khalifa, Hesham R. El-Seedi

**Affiliations:** 1 Department of Zoology, Faculty of Science, Menoufia University, Shebin El-Kom 32512, Egypt; 2 Department of Chemistry, Faculty of Science, Menoufia University, Shebin El-Kom 32512, Egypt; 3 Botany Department, Faculty of Science, Taibah University, Al Madinah 42353, Saudi Arabia; 4 Department of Chemistry, Quaid-i-Azam University, Islamabad 45320, Pakistan; 5 College of Food Science, Fujian Agriculture and Forestry University, Fuzhou 350002, China; 6 Pharmacognosy Group, Department of Pharmaceutical Biosciences, Uppsala University, Biomedical Centre, SE 751 24 Uppsala, Sweden; 7 Department of Pharmacognosy, College of Pharmacy, King Saud University, Riyadh 11451, Saudi Arabia; 8 School of Food and Biological Engineering, Jiangsu University, Zhenjiang 212013, China; 9 Department of Molecular Biosciences, The Wenner-Gren Institute, Stockholm University, SE 106 91 Stockholm, Sweden; 10 International Joint Research Laboratory of Intelligent Agriculture and Agri-Products Processing, Jiangsu Education Department, Jiangsu University, Nanjing 210024, China; 11 International Research Center for Food Nutrition and Safety, Jiangsu University, Zhenjiang 212013, China

The journal *Metabolites* retracts the article “*In Vitro* Induction of Apoptosis in Isolated Acute Myeloid Leukemia Cells: The Role of *Anastatica hierochuntica* Methanolic Extract” [1], which is cited above.

Following publication, concerns were brought to the attention of the Editorial Office regarding the integrity of an image presented in this article [1].

Adhering to our complaint procedure, an investigation was conducted by the Editorial Office and Editorial Board, with input from the National Board for Assessment of Research Misconduct of Sweden, which confirmed indications of inappropriate editing within several panels presented in Figure 7. The authors did not provide a comment or supporting material for Editorial Board evaluation. Consequently, the Editorial Board has lost confidence in the reliability of the findings and decided to retract this publication [1], as per MDPI’s retraction policy (https://www.mdpi.com/ethics#_bookmark30).

This retraction was approved by the Editor-in-Chief of the journal *Metabolites.*

The authors did not provide a comment on this decision.
